# Macrophages in organ fibrosis: from pathogenesis to therapeutic targets

**DOI:** 10.1038/s41420-024-02247-1

**Published:** 2024-12-04

**Authors:** Yuanyuan Jiang, Rong Cai, Yu Huang, Like Zhu, Long Xiao, Caihong Wang, Lihong Wang

**Affiliations:** 1 Translational Medical Innovation Center, Zhangjiagang TCM Hospital Affiliated to Nanjing University of Chinese Medicine, Zhangjiagang, 215600 Jiangsu China; 2 Department of Obstetrics and Gynecology, Zhangjiagang Hospital Affiliated to Soochow University, Zhangjiagang, 215600 Jiangsu China

**Keywords:** Chronic inflammation, Inflammatory diseases

## Abstract

Fibrosis, an excessive self-repair response, is an age-related pathological process that universally affects various major organs such as the heart, liver, kidney, and lungs. Continuous accumulation of pathological tissue fibrosis destroys structural integrity and causes loss of function, with consequent organ failure and increased mortality. Although some differences exist in the triggering mechanisms and pathophysiologic manifestations of organ-specific fibrosis, they usually share similar cascading responses and features, including chronic inflammatory stimulation, parenchymal cell injury, and macrophage recruitment. Macrophages, due to their high plasticity, can polarize into different phenotypes in response to varied microenvironments and play a crucial role in the development of organ fibrosis. This review examined the relationship between macrophages and the pathogenesis of organ fibrosis. Moreover, it analyzed how fibrosis can be modulated by targeting macrophages, which may become a novel and promising therapeutic strategy for fibrosis.

## Facts

- Macrophages are considered to be crucial candidates involved in the complex mechanisms of organ fibrosis.
- Macrophages modulate the fibrotic process by producing a variety of soluble mediators such as TGF-β1.
- As macrophages play different roles in different microenvironments and stimuli, understanding the mechanisms can help guide clinical treatment of fibrosis.

## Open Questions

- What is the specific molecular mechanism by which different macrophage phenotypes mediate the occurrence of organ fibrosis?
- How can organ fibrosis be alleviated by altering macrophage phenotypes?
- What are the potential problems and challenges associated with targeting macrophages in the treatment of organ fibrosis?

## Introduction

Fibrosis is initiated by uncontrolled tissue regeneration processes, characterized by sustained activation of fibroblasts and tissue dysfunction. It is a major cause of organ failure in many chronic diseases and age-related conditions [1]. In fact, fibrosis is an essential stage in the tissue repair response after organ injury. When sustained inflammatory stimulation or severe injury occurs, the repair response is dysregulated, leading to excessive accumulation of extracellular matrix (ECM) and impairment of organ structure and function [2]. The symptoms of fibrosis are related to the tissues in which they are appear. Skin fibrosis generally causes skin hyperplasia, distortion, and even pain in some patients. Fibrosis in internal organs such as the heart, liver, kidney and lungs can lead to organ dysfunction and eventually death [3]. Excessive fibrosis has been identified as the primary cause of approximately 45% of deaths in developed countries, thereby imposing a significant burden on global healthcare systems [4]. However, there is no complete cure for fibrosis without harmful consequences. Therefore, it is necessary to develop safe and effective treatments.

Macrophages can be polarized into different phenotypes to perform various functions and play a crucial role in maintaining tissue homeostasis [5]. They are involved in all stages of wound healing after injury. Initially, macrophages exhibit a pro-inflammatory phenotype, which initiates the inflammatory response. Subsequently, they transform into a wound-healing phenotype that promotes fibroblast activation and ECM deposition, contributing to the repair of normal tissue structure [6]. As macrophages contribute to tissue repair, their essential role in the pathophysiology of fibrosis is also emerging, presenting promising therapeutic opportunities for related diseases. This review focuses on recent advances in understanding the role of macrophages in fibrosis. First, this review outlines the potential molecular mechanisms of fibrosis formation. Then, the origin and phenotype of macrophages, as well as the pathological mechanism by which they mediate fibrosis, are summarized. It also explores the relationship between macrophages and fibrosis in some important organs, including the heart, liver, kidney, and lung. Finally, it examines the potential directions and challenges of macrophage-targeted therapy for organ fibrosis, suggesting some ideas for the future treatment of fibrosis-related diseases.

## Molecular mechanisms of fibrosis

Fibrosis is a pathological process in which extracellular matrix proteins accumulate excessively at sites of inflammation or damaged tissue, and is associated with the majority of progressive chronic diseases [7]. Typically, myofibroblasts at the site of minor or transient tissue damage initiate a wound-healing program, generating temporary ECM that accelerates tissue repair, which is beneficial to the organism [8]. However, when the injury is severe or frequent, myofibroblasts are continuously activated, disrupting the normal wound-healing process and leading to an excessive accumulation of ECM [9]. Simultaneously, several inflammatory cells in damaged tissues, such as neutrophils and macrophages, are activated, releasing cytokines and growth factors that accelerate myofibroblast activation and ECM deposition [10]. Persistent or uncontrolled fibrosis disrupts the physiological structure and function of normal organs and tissues, eventually leading to permanent damage [11].

**Fig. 1:**
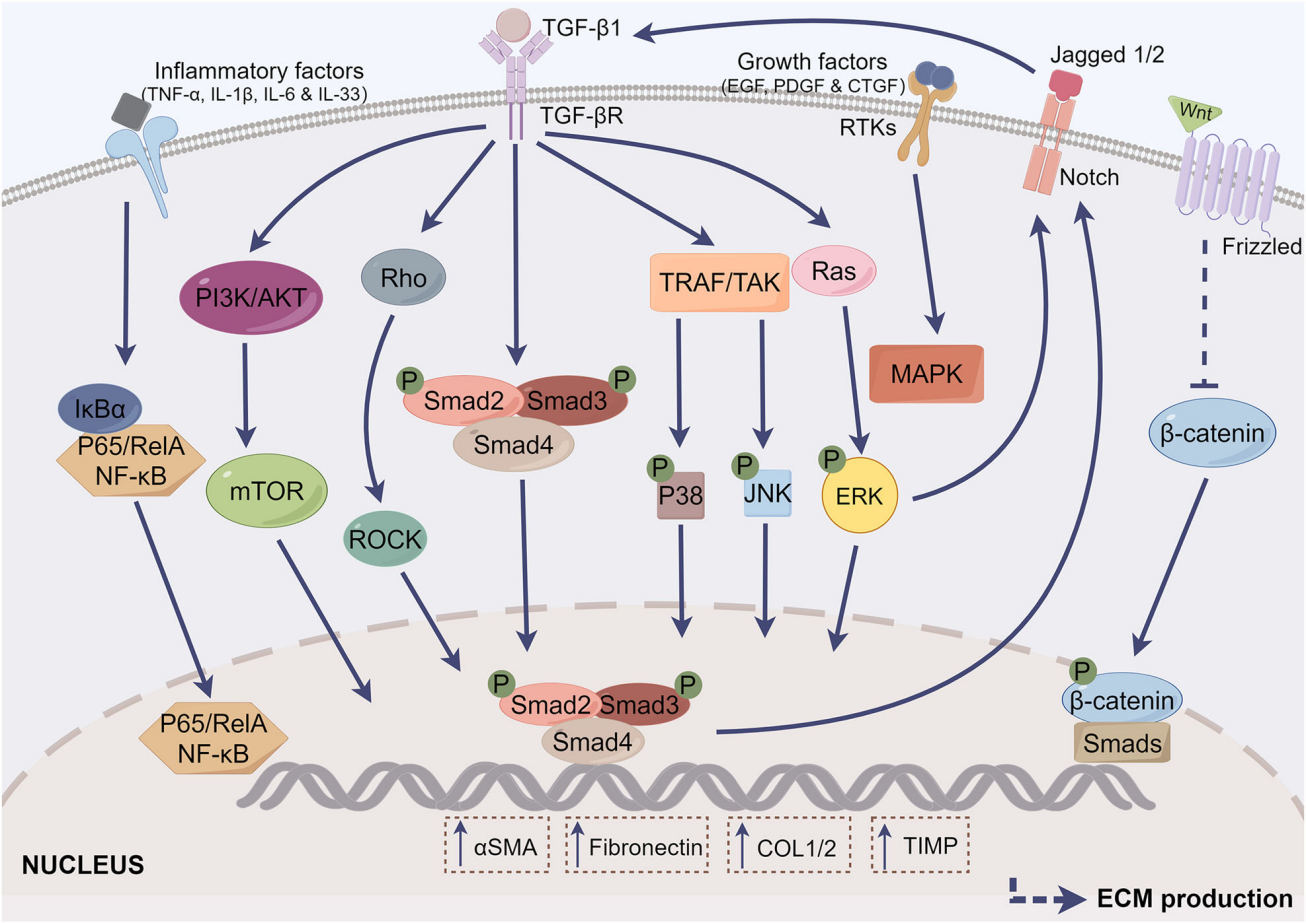
Molecular mechanisms of tissue fibrosis. The TGF-β/Smad signaling pathway is a classic pathway mediating tissue fibrosis. When TGFβ interacts with other downstream factors, such as PI3K, Rho, TRAF, and Ras, a non-classical pathway is initiated. As illustrated in the figure, inflammatory factors (TNF-α, IL-1β, IL-6, and IL-33), growth factors (EGF, PDGF, CTGF), the Wnt, and the Notch pathways are also involved in the regulation of tissue fibrosis. The activation of these signaling pathways induces the transcription of pro-fibrotic genes (α-SMA, fibronectin, COL1/2, and TIMP), which ultimately leads to the activation of myofibroblasts and the production of the ECM. This figure was drawn using Figdraw.

Fibrosis is commonly observed in multiple aging-related tissues and organs such as the heart, liver, kidney, and lung, and is often accompanied by chronic inflammation [12]. The pathological mechanisms of fibrosis are diverse in different tissues and organs, and they mainly include chronic inflammation, aging, and gene regulation [13]. Fibrosis is typically a synergistic process involving inflammatory factors, activation of fibroblast effector cells, and accumulation of ECM proteins. However, the mechanisms are complex, and the transformation of fibroblasts into myofibroblasts is a key driver of all forms of fibrosis [14]. Myofibroblasts are activated forms of fibroblasts that usually have two distinctive characteristics: a. contractions caused by the expression of α-smooth muscle actin (α-SMA); b. the ability to secrete ECM macromolecular proteins, such as fibronectin, type I collagen (COL1), type II collagen (COL2), and tissue inhibitors of metalloproteinase (TIMP) [14]. Organ fibrosis is associated with damage to parenchymal cells, typically accompanied by the recruitment of immune cells and subsequent inflammatory responses. Sustained chronic inflammation leads to the secretion of various cytokines by immune cells, including transforming growth factor beta (TGF-β), platelet-derived growth factor (PDGF), and interleukins (ILs), which mediate the excessive production of ECM [12]. To date, several mediators are involved in fibroblast activation by regulating downstream signaling pathways and promoting fibrosis progression (Fig. 1). The TGF-β superfamily is considered an important mediator for tissue repair, with different isoforms playing distinct roles in wound healing. Among them, TGF-β1 is the most critical regulator of tissue fibrosis, primarily inducing the transcription of pro-fibrotic genes by activating the downstream small mother against decapentaplegic (Smad) signaling pathway. The Smad family is primarily categorized into three types: receptor-activated Smads (R-Smads), inhibitory Smads (I-Smads), and co-partner Smads (Co-Smads). Upon binding to its specific receptors, TGF-β1 initiates signaling cascades that activate downstream R-Smad, specifically Smad2 and Smad3. These phosphorylated R-Smads subsequently form complexes with the Co-Smad known as Smad4. These complexes are then translocated into the nucleus, where they modulate the expression of pro-fibrotic proteins [15]. Additionally, TGF-β1 can promote ECM deposition through Smad-independent signaling pathways, including extracellular signal-regulated kinase (ERK), phosphatidylinositol 3-kinase/protein kinase B (PI3K/AKT), c-Jun N-terminal kinase (JNK), p38, and Rho-like small GTPases [16]. Simultaneously, some growth factors, such as epidermal growth factor (EGF), PDGF, and connective tissue growth factor (CTGF), can signal through receptor tyrosine kinases (RTKs) to mediate the activation of the mitogen-activated protein kinase (MAPK) pathway, which cooperatively regulates the TGF-β response [17, 18]. Wnt/β-catenin signaling cascades have also been associated with tissue fibrosis [19]. Rajasekaran et al. demonstrated that enhanced Wnt signaling was accompanied by increased expression of fibrotic marker proteins [20]. The transformation of non-motile epithelial cells into mesenchymal cells with invasive and motile properties is known as epithelial-mesenchymal transition (EMT), which increases ECM deposition and accelerates the progression of fibrosis under pathological conditions [21]. Lineage tracing studies have found that activation of the Notch signaling pathway significantly enhances EMT response [22]. Moreover, the Notch signaling pathway has been revealed to cross-talk with the TGF-β/Smad pathway, which jointly participates in the regulation of EMT and tissue fibrosis [23]. It is worth noting that tissue damage, repair, and fibrosis are often accompanied by the participation of several inflammatory cells [24]. Some inflammatory factors, such as ILs (IL-1β, IL-6, and IL-33) and tumor necrosis factor-α (TNF-α), activate the downstream nuclear factor-kappaB (NF-κB) signaling pathway, which regulates the expression of genes involved in fibrosis [25–28]. Consequently, regulating the production and secretion of these fibrotic mediators may be a potential strategy for anti-fibrotic therapy.

## Macrophages

### Origin of macrophages

**Fig. 2:**
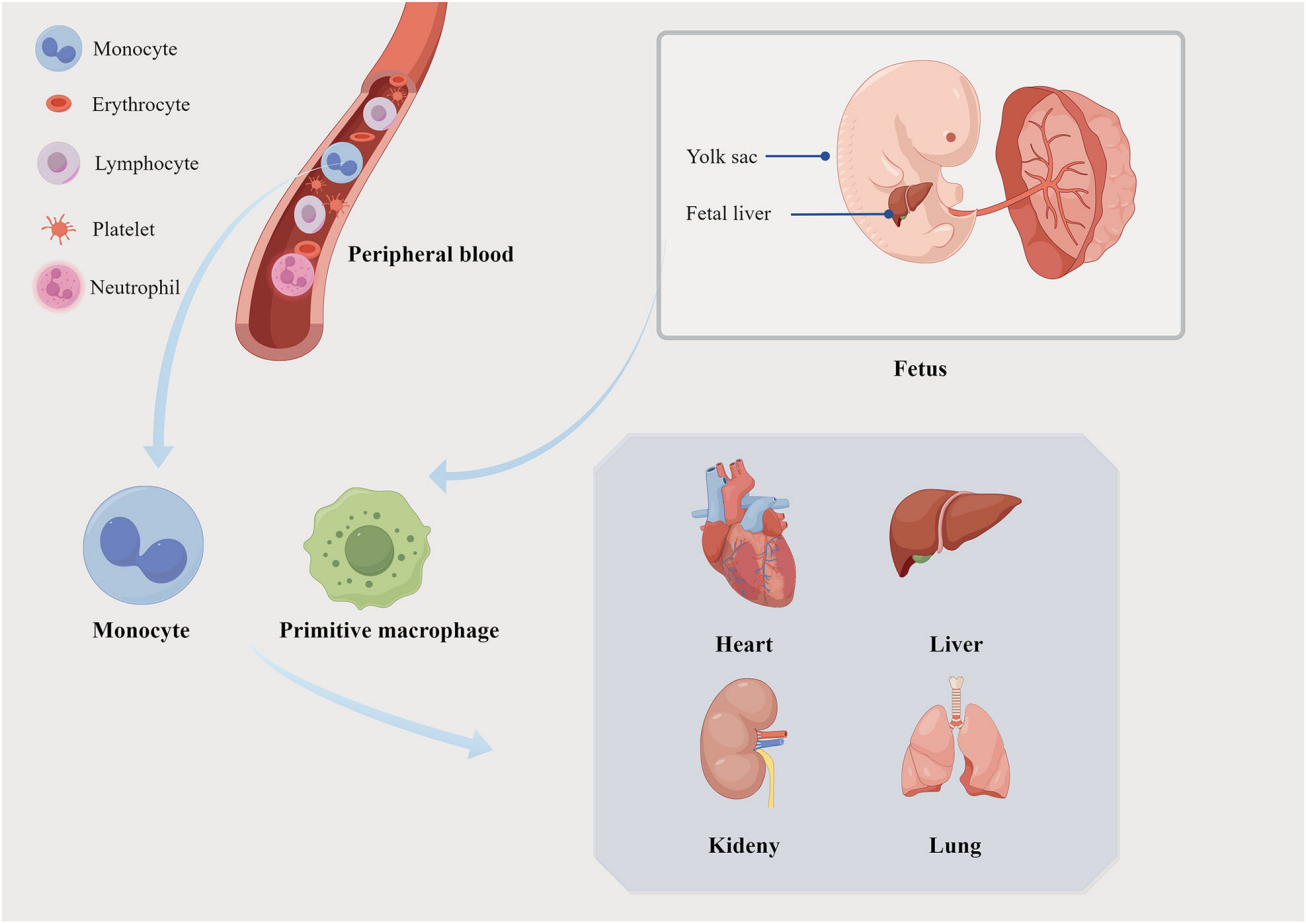
Origin of macrophages in different organs. Macrophages in organs are derived from monocytes in the peripheral blood and yolk sac/fetal liver. After tissue injury, monocytes or primitive macrophages further differentiate into different macrophage types under the stimulation of different inflammatory factors, mediating tissue inflammation and repair. Some of the information presented in this figure is sourced from ref. [35]. This figure was drawn using Figdraw.

Macrophages are an important part of the mononuclear phagocytic system (MPS) and are mainly derived from monocytes in blood circulation [29]. However, tissue macrophage biology has remained the focus of attention in recent years, particularly in terms of the establishment of tissue macrophage populations during organism development. It has been speculated that macrophages in organs are not only derived from monocyte differentiation but that some are seeded before birth [30]. In fact, studies have found the presence of a large number of macrophages during early development independent of blood monocytes [31]. These macrophages, which usually can self-renew, originate from the embryonic yolk sac and are known as tissue-resident macrophages [32]. Normally, tissue-resident macrophages locally self-expand and renew to maintain tissue homeostasis, although the ontogeny of tissue-resident macrophages in various human organs is currently unclear. It has been reported that the number of macrophages in the tissues of patients with monocyte deficiency is largely unaffected, providing additional evidence for this viewpoint [33]. In fact, tissue-resident macrophages exist in almost all tissues and organs, and they are classified into different subtypes according to their anatomical location and phenotypes, such as heart macrophages in the heart, Kupffer cells in the liver, kidney Macrophages in the kidney, and alveolar macrophages in the lung [34]. Under inflammatory or pathogenic conditions, many circulating monocytes in the blood are recruited to replenish the tissue-macrophage compartment [35]. Circulating monocytes have been reported to be induced by chemokines to migrate to inflammatory tissues and differentiate into inflammatory macrophages, which are involved in the regulation of the inflammatory response and tissue repair [36]. Therefore, tissue macrophages are mainly composed of a heterogeneous population of embryonically originated tissue-resident macrophages and inflammatory macrophages that have differentiated from blood monocytes (Fig. 2).

## Macrophage phenotypes

**Fig. 3:**
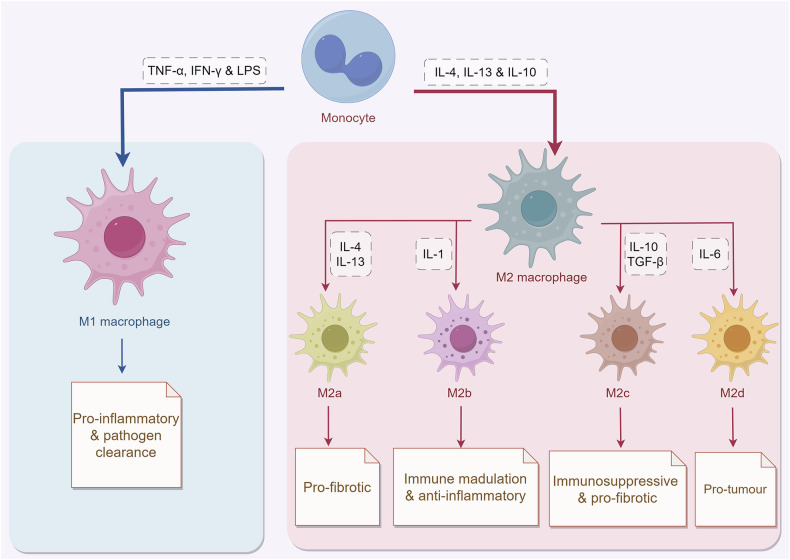
Functional plasticity of macrophages. Monocytes can selectively differentiate into classical M1 and M2 macrophages in different microenvironments. Under different cytokine stimulation, M2 macrophages can be divided into M2a, M2b, M2c and M2d subtypes. This figure was drawn using Figdraw.

Macrophages are characterized by high plasticity and diversity, exhibiting distinct phenotypes and functions in different pathophysiological tissue microenvironments (Fig. 3). Generally, macrophages are classified into two polarized phenotypes: pro-inflammatory (M1) and anti-inflammatory (M2) [37]. M1 macrophages differentiate in response to Th1-related cytokines (TNF-α and interferon-γ (IFN-γ)) or bacterial lipopolysaccharide (LPS) stimulation, which can secrete higher levels of inflammatory factors, including IL-1, IL-6, IL-12, IL-23, cyclooxygenase-2 (COX-2), and chemokine (C-C motif) ligand 8 (CCL8) to accelerate the inflammatory response. They are also involved in the activation of the nicotinamide adenine dinucleotide phosphate (NADPH) oxidase system, which induces reactive oxygen species (ROS) production and exerts antimicrobial and antitumor activities [38]. In contrast, M2 macrophages are induced by Th2 cytokines such as IL-4 and IL-13, as well as other anti-inflammatory cytokines such as IL-10 [39]. These macrophages are functionally anti-inflammatory and promote tissue repair by producing large amounts of immunosuppressive factors such as IL-10, arginase 1 (Arg-1), and TGF-β [40]. Moreover, M2 macrophages are further divided into four subtypes, M2a, M2b, M2c, and M2d, according to the stimuli they receive and the functions they perform [39]. M2a, which is activated by IL-4 and IL-13, is the most common M2 macrophage. It is pro-fibrotic and mainly secretes TGF-β, fibronectin, insulin-like growth factor (IGF), and chemokines (CCL1 and CCL12) [41]. M2b (IL-1 induced) are known as regulatory macrophages that help regulate inflammation and immune response [42]. IL-10 and TGF-β induce differentiation of M2c isoform with strong immunosuppressive and pro-fibrotic functions [42]. Moreover, there is a more harmful subtype (M2d), which is generally known as tumor-associated macrophages (TAMs). They can contribute to angiogenesis and the development and invasion of cancer cells [42].

## Role of macrophages in organ fibrosis

**Fig. 4:**
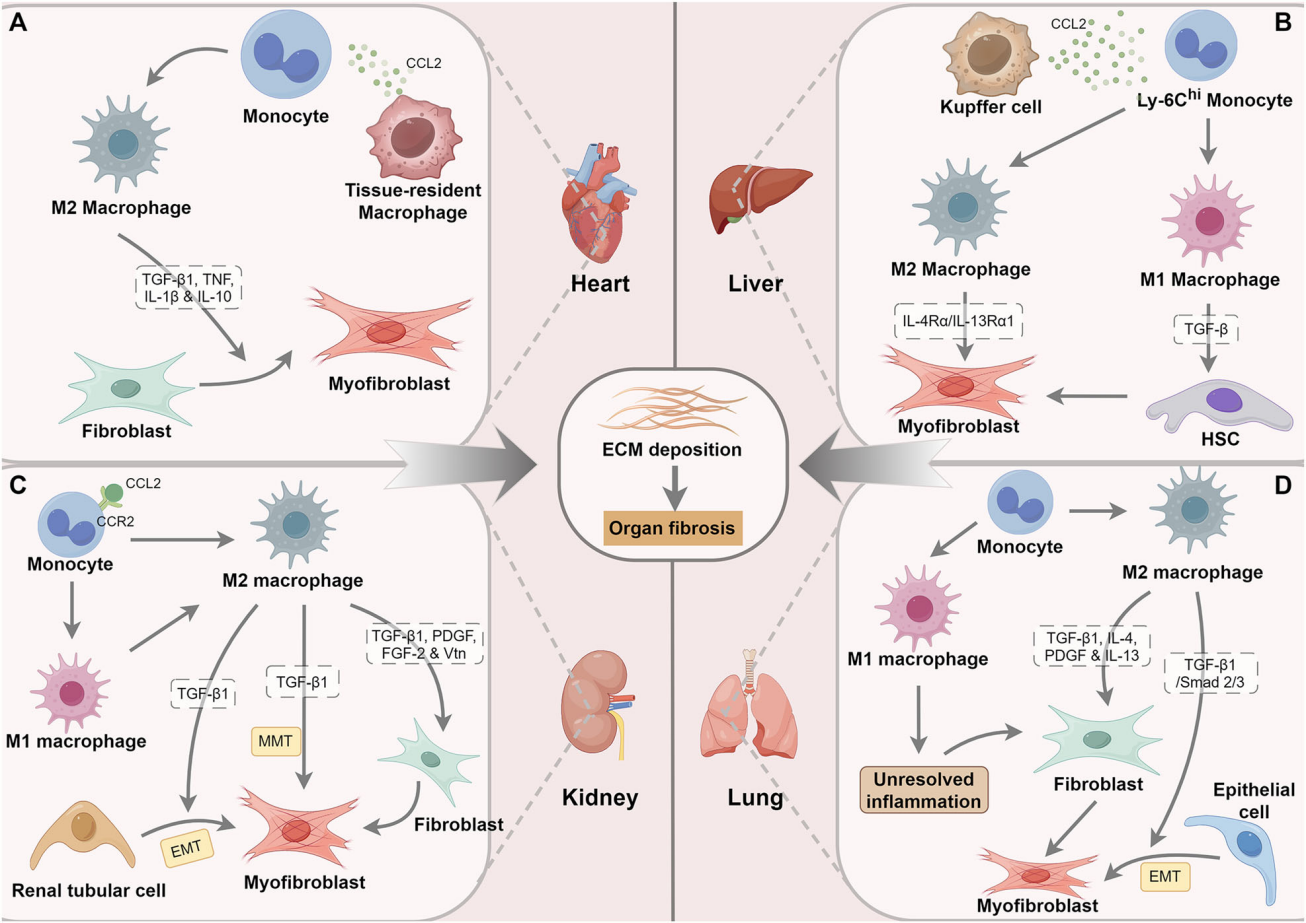
The role of macrophages in organ fibrosis. **A** Tissue-resident macrophages release inflammatory mediators, such as CCL2, to recruit circulating monocytes after cardiac injury. M2 macrophages derived from monocytes are crucial cells that induce cardiac fibrosis, mainly promoting the activation of myofibroblasts by secreting TGF-β1, IL-1β, TNF, and IL-10. **B** When liver cells are damaged, Kupffer cells present in the hepatic sinusoids release chemokines such as CCL2, recruiting Ly-6C^hi^ monocytes. Freshly infiltrated monocytes differentiate into M1 macrophages, which induce HSC activation by secreting TGF-β. The degree of liver fibrosis is primarily proportional to the number of M2 macrophages, which promote the production of ECM by myofibroblasts, mainly through the IL-4Rα/IL-13Rα1 pathway. **C** Renal cell injury triggers massive release of local CCL2 to recruit CCR2+ monocytes. In the later stages of damage repair, monocytes or M1 macrophages polarize toward M2 macrophages and promote myofibroblast transformation by secreting a variety of cytokines that mediate EMT, MMT, and fibroblast activation. **D** Injury events lead to the aggregation of monocytes in lung tissue. Then, monocytes further polarize into M1 and M2 macrophages. M1 macrophage-induced inflammation that has not been properly resolved leads to abnormal proliferation and activation of fibroblasts. M2 macrophages promote the transformation and activation of myofibroblasts by secreting pro-fibrotic factors and mediating the activation of the TGF-β/Smad2/3 pathway, which leads to excessive ECM and pulmonary fibrosis.

Fibrosis is the ultimate pathway for chronic diseases in a variety of organs, including the heart, liver, kidneys, and lungs [43]. Severe deterioration of these organs can eventually lead to death [44]. Age-related oxidative stress, chronic inflammation, and organ dysfunction are also important factors that can trigger and intensify tissue fibrosis [45]. Obviously, inflammation is closely linked to the development of age-related diseases. The quantity and distribution of macrophages within organs and tissues play a crucial role in affecting tissue repair and immune homeostasis [46]. Macrophages are also involved in mediating the progression of tissue fibrosis with age [47]. We then focused on the relationship between macrophages and age-related tissue fibrosis, including cardiac, liver, kidney, and idiopathic pulmonary fibrosis (Fig. 4).

### Cardiac fibrosis

Cardiac fibrosis is a common pathophysiological process occurring in most heart diseases and an important clinical feature of cardiac aging [48, 49]. Aging is a risk factor for various cardiovascular diseases and increases the mortality rate of heart-related diseases [50]. Importantly, cardiac fibrosis is one of the main predisposing factors for heart failure in older adults. Darband et al. found that age-induced cardiac fibrosis can be effectively improved by regulating the inflammatory process [51]. Therefore, effectively modulating the inflammatory response and inhibiting cardiac fibrosis may be a potential therapeutic strategy for preventing heart failure. Macrophages are considered key regulators of inflammation and cardiac fibrosis following myocardial tissue injury (Fig. 4A) [52]. In the homeostatic heart, tissue-resident macrophages predominate. However, during cardiac injury or stress, there is a marked increase in the population of cardiac macrophages, primarily due to the recruitment of monocytes mediated by CCL2, which is secreted by the tissue-resident macrophages [53]. Toba et al. identified that macrophage-derived matrix metalloproteinase-9 (MMP-9) significantly increased the cardiac inflammatory response, induced excessive ECM deposition, and aggravated age-dependent cardiac fibrosis [54]. Cardiac macrophages are usually found to be significantly expanded in hypertensive or aged mice, which indirectly induce fibroblast activation and ECM deposition by secreting IL-10 [55]. Although IL-10 is generally believed to be an anti-inflammatory agent that promotes wound healing, it also contributes to fibrosis in some aging-related chronic diseases [55]. Trial et al. demonstrated that M2 macrophages derived from circulating monocytes were more associated with the development of cardiac fibrosis than resident cardiac macrophages [56]. Human kallikrein 1 (hKLK1), a gene involved in tissue fibrosis prevention, can alleviate cardiac fibrosis and dysfunction by reducing macrophages recruitment and M2 macrophages polarization [47]. Using mass spectrometry, Shen et al. found that after myocardial ischemia (MI) reperfusion, TGF-β levels were significantly reduced in the cardiac tissues of macrophage-depleted mice, and cardiac fibrosis was significantly alleviated [57]. A recent study that integrated multiple MI scRNA-seq datasets further revealed that macrophages regulate myofibroblast activation through the secretion of TGF-β1, TNF, and IL-1β, which are associated with myocardial infarction fibrosis [58]. Furthermore, Zhuang et al. established that macrophages contribute to the progression of cardiac fibrosis and that macrophages derived from the peripheral mononuclear system are more committed to the transformation to myofibroblasts [59].

### Liver fibrosis

Liver fibrosis, is the result of chronic liver injury caused by various factors. Its pathogenesis involves the excessive accumulation of ECM due to the activation of hepatic stellate cells (HSCs) [60]. Inadequate intervention after the occurrence of liver fibrosis will lead to hepatocellular dysfunction, cirrhosis, and even liver cancer [61]. It has been demonstrated that aging is closely related to abnormal liver function and changes in liver structure. It is one of the important pathogenic factors leading to liver fibrosis and various liver diseases [62]. Delire et al. revealed that, compared to younger mice (aged 7 weeks), older mice (aged 15 months) exposed to carbon tetrachloride (CCl4) under the same conditions exhibited more severe liver fibrosis [63]. Macrophages are ubiquitous in the liver and are essential for liver homeostasis and the age-related progression of liver fibrosis [64]. Hepatic macrophages are primarily divided into resident macrophages, known as Kupffer cells, and circulating monocyte-derived macrophages (Fig. 4B) [65]. Following hepatic injury, Kupffer cells located within the hepatic sinusoids become activated and subsequently secrete inflammatory mediators, such as TNF and IL-1β, which stimulate tissue inflammation. They also release chemokines, such as CCL2, to facilitate the recruitment of circulating monocytes to the site of damage [66]. These recruited monocytes can be categorized into two subsets: high-expressing Ly-6C (Ly-6C^hi^) monocytes and low-expressing Ly-6C (Ly-6C^lo^) monocytes. Furthermore, activated Kupffer cells differentiate into M1 macrophages and M2 macrophages in response to different environmental stimuli [67]. Gene expression analysis of Kupffer cells and macrophages derived from monocytes showed high expression of TGF-β, indicating that both cell types contribute to the progression of liver fibrosis [68]. Notably, there is a significant reduction in the number of Kupffer cells during the initial days following liver injury. Concurrently, Ly-6C^+^ macrophages, which are derived from Ly-6C^hi^ monocytes, emerge as the predominant liver macrophage population responsible for mediating inflammation and fibrosis [66]. Freshly infiltrated macrophages display pro-inflammatory phenotypes and promote the differentiation of HSCs into myofibroblasts through the secretion of TGF-β, leading to excessive ECM deposition and liver fibrosis [69]. Interestingly, studies have shown that an increased number of M2 macrophages in liver fibrosis is positively correlated with the severity of the disease [70, 71]. Moreover, M2 macrophages activation after *Schistosoma* infection induces the secretion of pro-fibrosis factor and promotes liver fibrosis through IL-4Rα/IL-13Rα1 signaling pathway [72]. Conversely, during fibrosis reversal, Weng et al. demonstrated that M2 macrophages can also activate MMP-12 through IL-4Rα signaling to mediate the regression of liver fibrosis [73]. This suggests that macrophage IL-4Rα signaling plays a role in the regulation of fibrosis in a context-dependent manner.

### Renal fibrosis

Renal fibrosis is a common pathological process associated with chronic kidney disease (CKD) and any persistent renal tissue repair [74]. Renal replacement therapies, such as dialysis and transplantation, are the primary clinical treatment options for patients with CKD progressing to end-stage renal disease; however, their limited accessibility poses a significant public health challenge worldwide [75]. The kidney is the organ most affected by aging and is particularly susceptible to aging-related impairments [76]. Patients with diabetic kidney disease have significantly lower kidney function with age [77]. Early cDNA microarray and proteomic analysis demonstrated that kidney aging is highly similar to CKD [78]. Therefore, inhibiting age-related renal fibrosis can help prevent and control multiple renal diseases. Macrophages exist in renal tissues in both normal and diseased states and play an important role in the progression of renal fibrosis through their unique phenotype and function [79]. During the initial stages of renal cell injury, a substantial release of chemokines, particularly CCL2, occurs to facilitate the recruitment and accumulation of CC chemokine receptor 2-expressing (CCR2+) monocytes, thereby initiating an inflammatory response (Fig. 4C) [80]. In general, monocytes/macrophages in the kidney exhibit two extreme phenotypes depending on the local tissue environment. On the one hand, M1 macrophages help clear infections and cause kidney damage; on the other hand, M2 macrophages play an important role in injury repair [81]. In the later stages of tissue repair, macrophages undergo polarization toward the M2 phenotype, which is characterized by the release of anti-inflammatory mediators. Several studies have identified that the accumulation of M2 macrophage subsets expressing CD206 and/or CD163 promotes the progression of renal fibrosis [82, 83]. M2 macrophages may contribute to the development of renal fibrosis through several mechanisms. They are capable of producing substantial amounts of pro-fibrotic factors, including TGF-β1, PDGF, and fibroblast growth factor 2 (FGF-2). These factors facilitate the activation of myofibroblasts and lead to the excessive accumulation of ECM [84]. Furthermore, high levels of TGF-β1 released by M2 macrophages can induce EMT response of renal tubular cells, thereby advancing the progression of renal fibrosis [85]. Simultaneously, prolonged activation of TGF-β1 prompted the conversion of M2 macrophages into myofibroblasts, a process referred to as the macrophage-myofibroblast transition (MMT) [86, 87]. Vitronectin (Vtn) is a glycoprotein that is widely expressed in fibrotic regions of various diseases and contributes to the composition of the fibrotic microenvironment [88]. A recent study has revealed that M2 macrophages can promote fibroblast activation to induce renal fibrosis by assembling an extracellular matrix microenvironment enriched with Vtn protein [88]. These findings also provide potential targets for renal fibrosis treatment.

### Idiopathic pulmonary fibrosis (IPF)

IPF is a highly prevalent and fatal lung disease characterized by respiratory failure caused by ECM protein deposition and myofibroblast proliferation in the lung interstitium [89]. Although the underlying pathogenic mechanisms of IPF are not been completely understood, aging is considered to be one of the important factors in promoting its occurrence and development [90]. IPF has been reported to affect more than three million people worldwide, with a significant increase in prevalence with age [91]. The ECM in the lung interstitium is primarily derived from myofibroblasts. The role of macrophages in pathogenic pulmonary fibrosis has received increasing attention in recent years due to their distribution near myofibroblasts and expression of fibroblast activators [92]. Interestingly, it has been demonstrated that the lungs of aged mice contain a more significant number of highly activated macrophages than those of young mice [93]. IPF is usually caused by abnormal wound healing due to recurring lung injury [94]. Osteopontin (OPN, SPP1) is a matrix protein significantly associated with pulmonary fibrosis and supports macrophage proliferation. Secreted SPP1 has been reported to be significantly overexpressed in IPF [95], whereas SPP1-deficient mice models of pulmonary fibrosis exhibit reduced expression of COL1 and MMP2, as well as reduced pulmonary fibrosis [96]. Using single-cell sequencing, Morse et al. found that highly expressing SPP1 (SPP1^hi^) macrophage subpopulation increased in human IPF, suggesting the potential pro-fibrotic role of these macrophages in IPF lungs [97]. Gibbons et al. also observed that, although lung macrophages are not involved in the early stages of pulmonary fibrosis (PF), their activation significantly contributes to the subsequent progressive fibrotic process [98]. Alveolar macrophages (AMs) are predominantly categorized into two distinct populations: tissue-resident macrophages and monocyte-derived macrophages. Typically, tissue-resident macrophages are integral to immune defense and the maintenance of microenvironmental homeostasis within pulmonary tissue. Upon damage to alveolar epithelial cells, a large number of monocytes are recruited into the lung interstitium to differentiate into AMs and gradually replace the functions of tissue-resident macrophages [99]. Subsequently, these AMs undergo further differentiation into two functionally distinct subtypes, M1 and M2, in response to varying stimuli (Fig. 4D). In the early stages of injury, AMs are primarily induced to differentiate into M1 macrophages, which mediate inflammatory responses. Then, these cells differentiate into M2 macrophages, which are essential for mediating anti-inflammatory responses and promoting wound healing. However, if the inflammatory response initiated by M1 macrophages is not adequately resolved, it may lead to exacerbated lung tissue damage, resulting in abnormal proliferation of fibroblasts and the excessive deposition of ECM [100]. M2 macrophages are recognized as the principal effector cells in promoting tissue repair and inducing PF, primarily through the secretion of pro-fibrotic factors such as TGF-β1, IL-4, PDGF, and IL-13, which stimulate fibroblast proliferation and myofibroblast activation [101]. Furthermore, the secretion of TGF-β1 activates Smad2/3 to facilitate EMT, which is also one of the ways M2 macrophages promote PF progression [102]. In summary, these data suggest that the function of macrophages in IPF pathology is dependent on the extracellular microenvironment.

## Therapeutic potential of macrophages in organ fibrosis

Tissue fibrosis is associated with several highly prevalent chronic diseases and increased mortality with age, making it an urgent public health concern. Although many strategies for the prevention and treatment of tissue fibrosis have been explored and developed, their efficacy remains unsatisfactory. Consequently, it is particularly necessary to investigate and propose potentially viable therapeutic approaches to eliminate fibrosis. Macrophages have received increasing attention in the context of aging and tissue fibrosis regulation due to their high plasticity and functional diversity in the tissue microenvironment. Thus, we highlight possible strategies and applications for targeting macrophages during the development of fibrosis in selected major organ tissues that are highly attractive for fibrosis treatment.

### Blocking recruitment and expansion

Blocking monocyte recruitment is a crucial strategy for inhibiting macrophage-induced organ fibrosis. For instance, Patel et al. using RS-504393 (a CCR2 antagonist) and MC21 (an anti-CCR2 monoclonal antibody) in the transverse aortic constriction (TAC) mice to demonstrate that blocking CCR2 signaling to reduce pro-inflammatory Ly6C^hi^CCR2^+^ monocyte accumulation contributed to alleviating cardiac fibrosis [103]. Similarly, in unilateral kidney ischemia/reperfusion injury (U-IRI) mice treated with the CCR2 antagonist RS102895, the number of renal macrophages was reduced, pro-fibrotic cytokine expression was decreased, and renal fibrosis improved [104]. Furthermore, a recent study reported that cenicriviroc (CVC), a dual CCR2 and CCR5 antagonist, was used in a phase IIb clinical trial involving 289 adult patients with nonalcoholic steatohepatitis (NASH) and fibrosis. The results indicated that the antagonist was effective in treating fibrosis [105]. Although fewer chemokine-inhibiting drugs are currently approved for the clinical treatment of fibrotic diseases, several antagonists/inhibitors targeting CCR2 or other chemokines (such as CCR5, CCR3, CCL2) have been developed and are undergoing partial clinical trials for certain metabolic diseases, including diabetic nephropathy-induced CKD [106], as well as other fibrosis-related diseases [107].

Macrophage colony stimulating factor 1 (M-CSF1) is an essential growth factor that regulates monocyte and macrophage infiltration, proliferation, and differentiation. Mainly, it signals by binding to CSF1 receptors (CSF1R) [108]. Some early studies indicated that macrophage depletion with anti-CSF1 or anti-CSF1R can alleviate the development of liver fibrosis in mice [109, 110]. Similarly, Meziani and colleagues found that monoclonal antibodies against CSF1R have anti-fibrotic effects in a radiation-induced mouse model of pulmonary fibrosis [111]. To date, several small-molecule inhibitors targeting the inhibition of the CSF1R signaling pathway have been clinically validated [112]. Overall, therapeutic strategies targeting chemokines or the CSF1/CSF1R axis aim to reduce inflammatory monocyte recruitment and accelerate depletion of tissue-resident macrophages and can be considered as potential therapeutic targets for alleviating tissue fibrosis.

### Altering macrophage function

In addition to changing the number of macrophages, recent research has focused on specifically targeting or reprogramming their function to enhance the therapeutic potential of macrophages in fibrosis. Several drugs that alter macrophage function have been proven to be effective in the treatment of cardiac, liver, renal, and pulmonary fibrosis (Table 1). Using bioinformatic analysis techniques to analyze the relative proportions of immune cell subtypes in myocardial tissue from multiple sets of heart failure and non-heart failure clinical samples, the investigators found that M2 macrophages may be the predominant macrophage subtype in cardiac fibrotic tissue [113]. In subsequent experiments, they found that sodium tungstate (NaW), a phosphatase inhibitor, can reprogram M2 macrophages to the appropriate number of M1 macrophages by altering their glycogen metabolism, thereby facilitating the suppression of cardiac fibrosis levels in MI model mice [113]. Given the critical role of pro-inflammatory macrophages in the progression of liver fibrosis, many anti-inflammatory drugs and active ingredients from traditional Chinese medicine have demonstrated potential in the treatment of this condition. For example, in a mouse model of CCl4-induced liver fibrosis, capsaicin inhibited fibrosis progression by inhibiting Notch signaling pathway activation and M1 macrophage polarization, thereby reducing the release of inflammatory factors [114]. One of the active ingredients of *Forsythia suspensa*, phillygenin (PHI), is hypothesized to suppress M1 macrophage polarization by blocking the janus kinase/ signal transducer and activator of transcription 1 (JAK/STAT1) and Notch pathways, which lead to the activation of HSCs and ECM deposition [115]. However, hydroxychloroquine (HCQ) was found to reprogram macrophage function in a dose-dependent manner in the unilateral ureteral obstruction (UUO) mouse model. HCQ at 50 µM concentration mainly reduced M1 macrophage activation, while 100 µM HCQ concentration mainly reduced M2 phenotype macrophages differentiation, inhibiting TGF-β1 signaling pathway-mediated renal fibrosis [116]. Empagliflozin, which was originally developed to treat diabetes, is a sodium-glucose transporter 2 (SGLT2) inhibitor [117]. The beneficial effects of Empagliflozin in chronic renal failure have gradually emerged in recent years, and it is hypothesized that empagliflozin may alleviate renal fibrosis by inhibiting the polarization of fibrosis-promoting M2 macrophages by regulating mitophagy and mTOR signaling pathways [117, 118]. Chen et al. used the purinergic receptor type Y, subtype 12 (P2Y12) inhibitor clopidogrel to block the MMT process induced by P2Y12-mediated TGF-β/Smad3 pathway activation, and renal fibrosis in UUO mice was significantly inhibited [119]. Similarly, recent studies on the treatment of PF have focused increasingly on the inhibition of M2 macrophage polarization. In mice with bleomycin (BLM)-induced PF, the administration of neotuberostemonine (NTS), an alkaloid derived from the root of *Stemona tuberosa Lour*, significantly reduced the number of inflammatory cells and the expression of alpha-SMA, TGF-β1, and collagen in lung tissue. Further studies revealed that NTS reduced the number of macrophages in lung tissue and inhibited M2 macrophages polarization to exert antifibrotic effects [120]. Additionally, pharmacological agents such as pirfenidone (PFD) [121], the natural flavonoid molecule Icariside II (ISE II) [122], and the traditional Chinese medicine *Schisandra chinensis fructus* [123] have been documented to alleviate PF by inhibiting the polarization of M2 macrophages.

**Table 1:** Summary of targeted modulation of macrophage function for the treatment of organ fibrosis.

Fibrosis type	Treatment	Models	Effects	References
Cardiac fibrosis	Sodium tungstate (NaW)	MI mouse model	Transformed the M2 phenotype into the anti-fibrotic M1 phenotype	[113]
Liver fibrosis	Capsaicin	CCl4 mouse model	Inhibited M1 macrophage polarization and TNFα secretion	[114]
	Phillygenin (PHI)	/	Inhibited M1 macrophage polarization and reduce HSCs activation	[115]
Renal fibrosis	Hydroxychloroquine (HCQ)	UUO mouse model	Reduced macrophage activation	[116]
	Empagliflozin	5/6 nephrectomies rat model	Inhibited M1 and M2 macrophage polarization	[117, 118]
	Clopidogrel	UUO mouse model	Inhibited activation of pro-fibrotic macrophage	[119]
Pulmonary fibrosis	Neotuberostemonine (NTS)	BLM-induced mouse model	Blocked the MMT process	[120]
	Pirfenidone (PFD)	Radiation-triggered mouse model	Decreased macrophage recruitment and inhibit M2 polarization	[121]
	Icariside II (ISE II)	BLM-induced mouse model	Inhibited M2 polarization	[122]
	*Schisandrae chinensis fructus*	BLM-induced rat model	Inhibited M2 macrophages polarization	[123]

While these studies indicate that macrophages present a promising target for fibrosis treatment, the precise modulation of their functions poses significant challenges. It is essential to inhibit the role of macrophages in promoting organ fibrosis while simultaneously preserving their capacity to combat microbial infections, modulate immune responses, and facilitate tissue repair. Interventions must account for the specific targeting of macrophage subsets, as well as the optimal dosage and timing of administration. Furthermore, it is important to acknowledge that animal models may not fully replicate the complexities of human diseases. Human patients exhibit greater heterogeneity than animal models with respect to genetic background, age, and drug toxicity. While various JNK inhibitors are postulated to exert anti-fibrotic effects in animal models by inhibiting macrophage polarization [124, 125], a phase II clinical trial utilizing the JNK inhibitor CC-930 for the treatment of patients with IPF was discontinued due to the occurrence of acute liver injury [126]. Encouragingly, a recent clinical trial assessing the efficacy and safety of CC-9001, another JNK inhibitor, in a cohort of 165 patients with IPF has produced positive results [127]. Therefore, any therapeutic strategy must undergo well-designed prospective clinical trials before clinical application. Furthermore, Galectin-3 (Gal-3) has been identified as a potential target for modulating macrophage function in various organ fibrosis diseases [128]. The Gal-3 inhibitor, GB0139, has demonstrated safety, efficacy, and tolerability in a phase I/IIa clinical trial involving healthy volunteers and patients with IPF [129]. Additionally, other Gal-3 inhibitors, such as GB1211 [130], are currently undergoing clinical trials. These promising results may pave the way for future clinical applications.

## Conclusions and perspectives

Fibrosis is a common complication of various chronic diseases that affect the function of major organs in the body. Moreover, it poses a burden on the global healthcare system, which cannot be disregarded. However, there are no effective drugs that can inhibit or reverse fibrosis. Although some pro-fibrotic signaling inhibitors, such as nintedanib, have been approved for IPF, they only improve patient survival without resolving tissue fibrosis [131]. Nonetheless, continued exploration of the mechanisms of organ fibrosis will make effective antifibrotic therapies possible in the future.

Macrophages are crucial immune cells that maintain tissue homeostasis and can be recruited and activated by many different mechanisms. As explored in this review, macrophages are considered important candidates for regulating tissue fibrosis mechanisms. Extended duration or excessive aggregation of pro-inflammatory M1 macrophages and/or sustained activation of pro-fibrotic M2 macrophages can cause an imbalance of damaged tissue through multiple signaling pathways, leading to the accumulation of connective tissue. As summarized in this investigation, tissue fibrosis can be ameliorated by reducing monocytes and macrophages recruitment and/or altering existing macrophage function, as reported in previous studies. However, it is debatable if macrophages are also involved in fibrotic resolution under certain circumstances. Consequently, it is essential to accurately understand the different phenotypes of the pro- and anti-fibrotic effects of macrophages, and to identify the appropriate microenvironment and stimuli.

Although there may be an overlap in the role of macrophages in regulating tissue inflammation, repair regeneration, and fibrosis in different rodent models, the origin and function of resident macrophages in different organs are not fully understood. Experimental animal models have made considerable progress in understanding the complexity and plasticity of macrophage biology during tissue fibrosis development. However, animal models do not fully mimic human diseases, and they usually only represent the pathological state of organ fibrosis in response to specific stimuli. Compared with rodents, the effects of various internal and external factors, such as age, sex, infection, and drug treatment, in humans are more heterogeneous. Therefore, future studies should pay more attention to the differences between animal models and human disease to develop antifibrotic therapies that can be effectively applied to clinical practice.

It is apparent that the origin, differentiation, and polarization of macrophages determine their different functions in organ fibrosis. To better elucidate the role of macrophages in the development of fibrosis, additional studies on fibrosis in individual organs are required. Despite these challenges, scientists believe that the continued development of advanced technologies will provide a deeper understanding of the mechanisms of different macrophage subtypes involved in the progression and resolution of organ fibrosis and the development of effective therapies for fibrotic diseases.
